# Platelet-rich plasma in alopecia areata and primary cicatricial alopecias: A systematic review

**DOI:** 10.3389/fmed.2022.1058431

**Published:** 2022-11-24

**Authors:** Kasama Tejapira, Tanat Yongpisarn, Nawara Sakpuwadol, Poonkiat Suchonwanit

**Affiliations:** Division of Dermatology, Department of Medicine, Faculty of Medicine, Ramathibodi Hospital, Mahidol University, Bangkok, Thailand

**Keywords:** AA, immune-mediated alopecia, lichen planopilaris, LPP, non-scarring alopecia, PCA, scarring alopecia, hair loss

## Abstract

**Background:**

Immune-mediated alopecias (IMAs), a group of hair disorders associated with immunological reactions, remain a therapeutic challenge since available treatments are generally unfavorable with potential side effects. Platelet-rich plasma (PRP) has been recently proposed as a treatment option based on several limited-quality studies; however, there is no systematic evaluation of PRP efficacy on IMAs in the literature.

**Objective:**

To assess PRP’s effects in treating IMAs using a systematic review.

**Methods:**

Electronic searches were conducted using PubMed, Embase, Scopus, and Cochrane Library databases. A search strategy was designed to retrieve all studies exploring PRP in treating IMAs, including alopecia areata (AA) and primary cicatricial alopecias (PCAs). In addition, all randomized and non-randomized studies reporting subjective and/or objective outcomes of alopecia treatment with PRP were included.

**Results:**

Thirty-two studies were included, comprising 621 patients with AA and 19 patients with PCAs. PRP had superior efficacy as monotherapy in five studies, comparable to intralesional corticosteroids in six studies in AA treatment. In addition, in the analysis of PCAs, including lymphocytic and neutrophilic subtypes, PRP was efficacious in alleviating disease progression in nine studies.

**Conclusion:**

PRP is considered a promising treatment for AA and PCAs in patients who experienced unfavorable outcomes from conventional treatment. However, its clinical application remains to be standardized, and its recommendation as a treatment for IMAs could not be ascertained due to a lack of high-quality evidence.

**Systematic review registration:**

[https://www.crd.york.ac.uk/prospero/display_record.php?RecordID=353859], identifier [CRD42022353859].

## Introduction

Alopecia is a common dermatological disorder affecting the population worldwide. The condition is highly associated with psychological distress and impacts patients’ quality of life (1). Alopecia manifests varyingly and is categorized into non-cicatricial (non-scarring) and cicatricial (scarring) alopecias, which include several disorders (2). In non-cicatricial alopecia, hair follicle (HF) stem cells located in the bulge area are preserved with potential for regrowth. In contrast, they are irreversibly destroyed in the cicatricial subtype, leading to permanent alopecia (3, 4).

Immune-mediated alopecia (IMA) refers to hair loss disorders associated with immune responses involved in inflammation and autoimmunity to HFs. HF is an area of relative immune privilege. Several mechanisms, such as downregulating major histocompatibility complex (MHC) and expressing signals using type-1 transmembrane glycoprotein CD200, help protect HF from inflammatory insults (5, 6). Imbalances in the protective mechanism of HF, also called immune privilege collapse, are theorized to be the pathogenesis of IMAs (6–9).

Alopecia areata (AA) and primary cicatricial alopecias (PCAs) are two major subtypes of IMAs. AA is an autoimmune, non-scarring hair loss disorder histologically characterized by CD8+ and CD4+ T cells infiltrating the peribulbar area of anagen HFs (10–13). Because the inflammatory process of AA conserves stem cells, reversible hair loss can occur after AA subsides. In contrast, inflammation in PCAs mainly involves the hair bulge region, where HF stem cells locate, leading to the permanent destruction of HF and replacement with a scar (14–17). PCAs are classified based on the types of predominant inflammatory cell involvement into lymphocytic, neutrophilic, and mixed cell infiltrates (3).

Treatment modalities of IMAs aim to suppress the inflammatory response, prevent potential hair loss, and promote hair regrowth. Several therapeutic options have been introduced, such as topical and intralesional corticosteroids, systemic immunosuppressants, topical immunotherapy, lasers, and phototherapy, depending on IMA subtypes, degree of inflammation, disease stage, and relevant comorbidities (18–22). However, their therapeutic efficacy is still debated since treatment outcomes are generally unpredictable. Moreover, poor response, high recurrent rate, and potential side effects are frequently reported (23–25).

Recent advancements in understanding the pathogenesis of IMAs have accelerated the discovery of novel treatments. In recent years, the regenerative capability of platelet-rich plasma (PRP) has been used to treat several dermatological diseases. PRP is an autologous plasma preparation with concentrated platelet produced by centrifugation (26, 27). It comprises over 20 growth factors and cytokines, such as transforming growth factor (TGF), platelet-derived growth factors (PDGF), insulin-like growth factor (IGF), vascular endothelial growth factors (VEGF), epidermal growth factor (EGF), and fibroblast growth factor (FGF), playing a significant role in initiating tissue repair by releasing biologically active factors and immunomodulatory effect of innate and adaptive immune system (27–29).

Some studies have reported PRP’s efficacy in treating AA and PCAs with positive outcomes, with fewer side effects; others revealed the opposite. Given this inconclusive issue, it is essential to integrate and compare these findings in the secondary analysis. We aimed to assess PRP’s efficacy in treating AA and PCAs *via* a systematic review due to a lack of systematic evaluation of the therapeutic effects of PRP on IMAs.

## Methods

### Study design

The protocol was registered in PROSPERO (International Prospective Register of Systematic Reviews; no.CRD42022353859). The systematic review followed the Preferred Reporting Items for Systematic Reviews and Meta-analyses (PRISMA) guidelines. Electronic searches were conducted from the database’s inception to July 1, 2022, *via* PubMed, Embase, Scopus, and Cochrane Library databases. Using keywords and a controlled vocabulary, the search strategy was designed to retrieve all studies exploring PRP use in treating AA and PCAs. There were no restrictions on the language or publication period of the searches. Conference abstracts were excluded. Details of the search strategy are presented in Supplementary Table 1.

### Study selection

Each article was reviewed independently by two reviewers (KT and TY). Disagreements were resolved *via* discussion with a third reviewer (NS). We included all randomized and non-randomized studies that reported any subjective and/or objective treatment outcomes.

### Data extraction

Data were extracted from the included studies using a standardized format. The following data were collected: study type, study characteristics (authors, publication year, and study design), patient characteristics [diagnosis, number of patients, disease duration, previous treatment(s), and age], intervention(s), PRP protocol, investigations, objective and subjective assessment of hair growth, incidence of adverse effect(s), and follow-up duration. Corresponding investigators were contacted *via* email if there was missing data. Two independent reviewers extracted data (KT and TY), and discrepancies were resolved with the assistance of a third reviewer (NS).

### Quality assessment

Quality assessment was performed using Rob-2 and ROBINS-1 for randomized and non-randomized studies, respectively (30, 31). Risk-of-bias plots were created using Risk-of-bias VISualization (robvis) (32).

## Results

### Study characteristics

**FIGURE 1:**
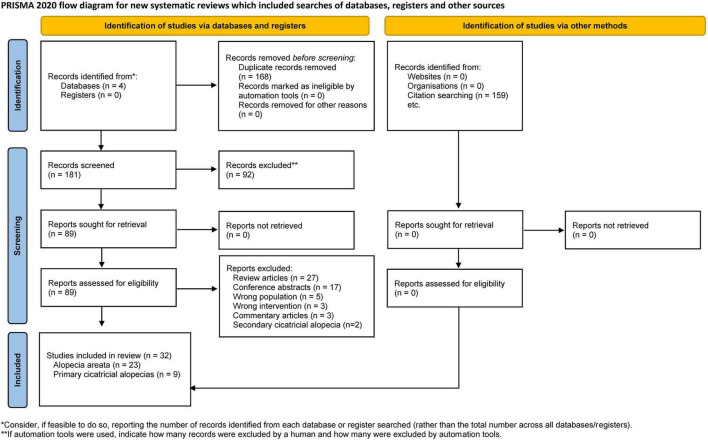
Flow diagram of search methodology and selection process based on the preferred reporting items for systematic reviews and meta-analyses (PRISMA) flowchart for the article selection process.

After removing duplicates, 181 papers were screened by title and abstract. At the full-text stage, 87 full articles met our predefined selection criteria, and we further excluded 55 publications for the following reasons: review articles (*n* = 27), conference abstracts (*n* = 17), wrong population (*n* = 5), wrong intervention (*n* = 3), commentary articles (*n* = 3), and secondary cicatricial alopecias (*n* = 2) (Figure 1). Thirty-two studies were included: 11 randomized controlled trials (RCTs) (33–43), 4 non-randomized studies (44–47), and 17 case series or case reports (48–64). Between 2013 and 2022, 23 AA studies (33–55) and nine PCA studies (56–64) were included, totaling 621 patients with AA and 19 with PCAs. Details of the included studies are summarized in Tables 1, 2.

**TABLE 1:** Characteristics of alopecia areata studies included in the systematic review.

References	Characteristic of enrolled subjects	Intervention	Objective measures	Objective assessment of hair growth	Subjective assessment of hair growth	Adverse effects	Follow up time
**Randomized control studies**							
Trink et al. (33)	Forty-five patients with chronic, recurring AA of at least 2 years duration, symmetrically distributed 4–6 patches of hair loss, 20 (44.44%) male, 25 (55.56%) female, mean age 28 years	● PRP injection ● TA injection (2.5 mg/ml) ● Placebo	● SALT score reduction ● Hair regrowth Trichoscopic evaluation (exclamation-mark hairs, black dots, yellow dots, and pigtail regrowing hair) ● Itching/burning sensation ● Disease relapse ● Ki-67 IHC staining	Trichoscopic exam ● 96% of PRP group, 25% of TA group had fully pigmented hair from the beginning of hair regrowth ● Both PRP and TA groups decreased the number of dystrophic hairs, but PRP led to significantly better dermoscopy results compared with TA treatment IHC ● PRP and TA significantly increased the levels of Ki-67 effect of PRP on Ki-67 levels was evident already after 8 weeks, and was sustained throughout the study period (1 year)	SALT score ● SALT score reduction in PRP group was significantly better than TA Hair regrowth ● 27% of TA group, 60% of PRP group achieved complete remission in 52 weeks Relapse ● 38% of TA group relapse at 24 weeks (no relapse in PRP group at 24 weeks) 71% of TA group, 31% of PRP group relapse at 52 weeks Itching and burning sensation ● PRP and TA decreased the itching or burning sensation of the patients	NR	52 weeks
El Taieb et al. (34)	Ninety patients with AA any severity, 49 (54.44%) male, 51 (56.67%) female mean age 21.20 ± 9.05 years	● PRP injection ● Topical 5% minoxidil ● Placebo	● Hair regrowth ● Dermoscopic exam (short vellus hair, yellow dots)	● Dermoscopic exam ● Both short vellus hair and yellow dots were significantly decreased after PRP treatment, significant increase in short vellus hair was seen in minoxidil and placebo	Significant hair regrowth was observed in ● Minoxidil group: 81% AA, 14% ophiasis, 5% AT ● PRP group: 70% AA, 30%AU ● Placebo group: 30% AA	NR	12 weeks
Nagaratna et al. (35)	Twenty-five AA patients with disease duration more than 24 weeks, Male:female 1.27:1, mean age 33.43 ± 7.22 years	● PRP ● No control reported	● SALT score ● Hair regrowth score	NR	SALT score ● Significant decrease in the SALT score from baseline (15.92 ± 2.07) to the end of 12 weeks (3.524 ± 2.11) (*p* < 0.05) Hair regrowth score ● 60.9% of the patients had > 50% hair regrowth at the end of 12 weeks	NR	36 weeks
Khademi et al. (36)	Ten patients with AT for at least 3 years, 5 (50%) men, 5 (50%) women, mean age 28.90 ± 6.28 years	● PRP ● NSS	● SALT score ● Hair regrowth	NR	SALT score ● Did not show any significant effect of PRP on changes of SALT score (*p* > 0.05) Hair regrowth ● No significant effect of PRP on hair regrowth was found (*p* > 0.05)	NR	16 weeks
Albalat and Ebrahim (37)	Eighty patients with chronic recurring AA 62/80 (77.5%) had < 25% scalp area involvement, 68 (85%) male, 12 (15%) female, mean age 34.29 ± 9.226 years	● PRP ● TA (5 mg/ml)	● SALT score (in 6-point sore, S0 = no alopecia −S5 = alopecia totalis) ● Hair regrowth score ● Dermoscopic photomicrograph ● Recurrence	Dermoscopic ● Both groups showed improvement in the number of pigmented hair and decreased percentage of dystrophic hairs (*p* < 0.001)	SALT score ● There was highly statistically significant improvement in SALT score after treatment in both intralesional TA and PRP groups, respectively (0.50 ± 0.75, 0.40 ± 0.71, *p* < 0.001) without statistically significant difference between groups (*p* = 0.49) Hair regrowth ● At 8 weeks it showed more improvement in hair regrowth score without significant difference between both groups Recurrence ● 2 (5%) in the PRP group vs. 10 (25%) in the ILC group showed recurrence	● No statistically significant difference was found between the two groups (mild erythema and pain) ● No serious side effects were detected	24 weeks
Ragab et al. (38)	Sixty patients with AA (excluded AT and AU), 48 (80%) male, 12 (20%) female, mean age 32.2 ± 13.098 years	● Intradermal PRP ● PRP with FCL ● PRP with micro needling	● Percentage of SALT score reduction ● Patient satisfaction ● Pain severity in visual analogue scale	NR	SALT score reduction and patient satisfaction ● No significant difference in the degree of improvement between the groups studied at the end of the sessions by physicians’ clinical assessment (*p* = 0.268) or by patient satisfaction (*p* = 0.147)	● Pain during the session was more obvious in intradermal injection > FCL > micro needling	24 weeks
Kapoor et al. (39)	Forty patients with Patchy AA on scalp with < 25% area involvement, 28/40 (70%) disease duration < 6mo, 18 (45%) male, 22 (55%) female. mean age 27.1 ± 7.07 years	● PRP ● TA (10 mg/ml)	● Percentage of SALT score reduction ● Pain severity in visual analogue scale ● Disease relapse	NR	Percentage of SALT score reduction ● Reduction in SALT score at each visit with respect to baseline was greater in the TA group [11/20 (55%) patient gained > 50% SALT score reduction] as compared to PRP group Disease relapse ● At 24 weeks after beginning of treatment 1/20 (5%) in TA group and 2/20 (10%) in PRP group got disease relapse	● The pain VAS score was statistically significant higher in PRP group (25.6 ± 10.65 vs. 2.25 ± 3.27)	24 weeks
Hegde et al. (40)	Fifty patients with patchy AA at scalp, age 18–60 years	● PRP ● Intralesional TA (10 mg/ml) ● Placebo	● Percentage of SALT score reduction ● Percentage change in dystrophic hair (assess in one representative alopecic patch between 12 and 3’O clock position) ● Hair regrowth	Percentage change in dystrophic hair ● No statistical significance improvement in dermoscopic findings between PRP and TA (*p* = 0.497) nor PRP and placebo (*p* = 0.448)	Percentage of SALT score reduction ● Statistically significant declined in mean SALT score was observed in all three groups by the end of 8 weeks (*p* < 0.001) Hair regrowth ● Statistically significant absolute growth and percentage regrowth were observed maximally in TA > PRP > placebo (*p* = 0.016 and 0.0108) ● Near complete regrowth after 3 session was observed in 11 (44%) of PRP-treated group and 10 (40%) of TA-treated group	● Pain during injection was observed in 18 (27%) of PRP-treated group and 5 (20%) of TA-treated group (*p* < 0.05)	20 weeks
Balakrishnan et al. (41)	Forty patients with patchy AA confined only at scalp majority of patients had single patch, 60% of patients had disease duration < 12 weeks, 27 (67.5%) male, 13 (32.5%) female, age ≥ 18 years	● PRP ● Intralesional TA (10 mg/ml)	● SALT score reduction ● Hair regrowth scale (patient assessment)	NR	SALT score reduction ● At 8 weeks, significant difference in the score reduction in PRP > TA group (*p* = 0.028) ● At 12 weeks, no statistically significant difference between two groups Hair regrowth ● No statistically significant difference in hair regrowth between PRP (excellent 12.5%, good 31.3%) and TA-treated group (excellent none, good 18.8%)	● No side effect was reported in intralesional TA group ● 3/20 (15%) patients in Pin PRP-treated site and RP group reported severe pain during injection	12 weeks
Gupta et al. (42)	Twenty-seven AA patients with SALT score ≥ 25%, mean disease duration 6 years or longer, 13 (48.14%) male, 14 (51.85%) female, mean age 23.89 ± 4.64 years	● PRP ● NSS (placebo)	● Percentage of SALT score reduction ● Percent reduction of dystrophic hair ● LIKERT score on hair regrowth ● Patient global assessment score ● Biopsy with IHC at PRP injected site	Dermoscopic evaluation ● 9 (64.3%) PRP-treated site and 10 (66.7%) placebo-treated site showed ≥ 50% reduction in dystrophic hair marker ● In PRP treated site, lesional T helper, T reg cytokine mRNA expression mean interferon gamma (*p* = 0.001), IL-17 (*p* = 0.009) mRNA expression decreased significantly and mean IL-10 (*p* = 0.049) FOXP3 (*p* = 0.011) mRNA expression increased significantly	Physician subjective ● Significant difference in percent SALT score reduction at 20th week between PRP (9.05 ± 36.48%) and placebo (4.99 ± 33.88%) treated area ● 6/27 (22.22%) patients had comparable hair regrowth in PRP-treated and placebo-treated 9/27 (33.33%) better response on PRP site, 3/27 (11.11%) better response on placebo site, 4/27 (14.81%) no hair regrowth in either site Patient global assessment ● 3 (11%) in PRP-treated site and 2 (7.4%) in placebo-treated site were reported ≥ 50% improvement	● 23/37 (85.2%) reported pain on injected site after procedure, resolved in 2–5 days	20 weeks
Tawfik et al. (43)	Thirty patients with chronic recurrent AA for a period of 2 years, resistant to other lines of treatment, 26 (86.67%) male, 4 (13.33%) females, mean age 28.8 ± 6.8 years	● PRP ● LLLT 3 times weekly ● NSS (placebo)	● Hair growth by digital photograph ● Hair density and hair diameter by fototrichogram ● Patient satisfaction 12 weeks after last session	Hair density and diameter ● PRP patches showed significant improvement in hair density (*p* = 0.007) and hair thickness (*p* = 0.002) ● LLLT patches showed significant improvement in hair density only (*p* = 0.02) ● None of patches in placebo group showed significant increase in hair density and thickness	Hair growth ● 11 (44%) PRP patches and 8 (32%) LLLT patches showed more than 75% improvement in hair coverage ● 10 (40%) PRP patches and 11 (44%) in LLLT patches Patient satisfaction ● Maximum degree of contentment was reported at PRP-treated area	● Temporary pain in PRP-treated sites ● 5/25 (20%) patients reported scalp tenderness after LLLT, resolved within 2 h	18 weeks
**Non-randomized studies**							
Singh (44)	Twenty patients with patches AA with disease duration at least 2 years, age 25–35 years	● PRP	● Disease relapse	● 1 patient had relapse disease	NR	No adverse event	52 weeks
Khan et al. (45)	Twenty patients with patches of alopecia duration at least 24 weeks, 12 (60%) male, 8 (40%) female, age 16–50 years	● PRP	● Hair regrowth ● Disease relapse	Disease relapse ● Relapse did not occur in any patient	Hair regrowth ● 6/20 (30%) patients (4 female and 2 male) had excellent response ● 5/20 (25%) patients (4 male and 1 female) with good response ● 4/20 (20%) patients (2 male and 2 female) showed fair response ● 5/20 (25%) (4 male and 1 female) poor response ● Significant hair growth was seen after 8 weeks of PRP treatment	● Mild pain was noted for 5–30 min at site of injection ● No other side effect was noted nor reported by any of the patients	36 weeks
Fayed et al. (46)	Forty-one patients with AA multiple bilateral and symmetrical patches of AA, disease duration less than 24 weeks, 32 (78.04%) male, 9 (21.95%) female, Mean age 26.68 ± 4.49 years	● PRP injection ● NSS injection	● SALT score change Divided patients into four group (S1 = 25% hair loss S2 = 25–49% hair loss S3 = 50–74% hair loss S4 = 75–99% hair loss) ● DQOL questionnaire	NR	SALT score ● Decrease > 50% in 13 (31.7%) PRP, 2 (4.9%) placebo and statistically significant difference between treatment side and placebo side (*p* = 0.002) ● Grade1 (S1) showed the best improvement 12/23 (52.3%) ● Grade2 (S2) 1/7 (14.3%) had hair regrowth ● No response in grade 3–4 ● 15 (36.6%) suffered a very large effect, after treatment became 6 (14.6%), 12 (29.3%) had moderate degree, after treatment became 7 (17.1%), 12 (29.3%) mild degree, became after treatment 11 (26.8%)	● 31 (75.6%) tolerable pain duration no more than 30 min ● 6 (14.6%) tenderness, burning sensation ● 100% erythema at injection site for few hours	20 weeks
Fawzy et al. (47)	Thirty-one patients with AA (excluded AT, AU), 23 (74.19%) male, 8 (25.80%) female, mean age 32.67 ± 11.30 years	● PRP injection ● TA injection (5 mg/ml)	● SALT score reduction ● Trichoscopic examination ● AA symptom impact	Trichoscopic exam ● Statistically significant improvement in trichoscopic findings at final evaluation when compared to baseline in both group A and B	SALT score reduction ● Final SALT score showed significant lower levels in both groups in comparison with baseline levels (*p* = 0.025 and *p* = 0.008)	NR	12 weeks
**Case series/case reports**							
Donovan (48)	A 41-year-old female with ophiasis AA and bipolar disorder	Intralesional PRP	Hair regrowth	NR	Hair regrowth ● Hair regrowth was noted by 4 weeks with robust regrowth of hair measuring 2.8 cm by 12 weeks	Mild tender on day of the procedure and 2 following days	12 weeks
Mubki (49)	A 22-year-old female with chronic diffuse AA for 5 years	● Left half intralesional TA (2.5 mg/ml) ● Right half 4 PRP treatment sessions were alternated with 4 TA treatment sessions at 2 weeks intervals	● Hair density ● Hair diameter ● Hair coverage	Hair coverage ● Changes in the overall scalp hair coverage was minimal (<25%) in both halves of the scalp, right half (TA and PRP) showed slightly better improvement	Hair density ● Both treatment modalities; right half and left half resulted in an increase in the number of terminal hairs as compared to the baseline (16 and 12%, respectively) ● Hair diameter ● Only the right half showed an increase in the mean hair shaft diameter (+35%) compared to a decline by (−4%) in the left half	NR	18 weeks
De Vasconcelos et al. (50)	A 43-year-old female with AA for 1 year	PRP injection	● Hair regrowth ● Dermoscopic exam	NR	● At the end of the three sessions, hair regrowth was observed in the clinical and dermoscopic exams	NR	9 weeks
Fonseka et al. (51)	● A 25-year-old female with AA ● A 23-year-old female with AT ● A 55-year-old female with AT	PRP injection with 5% minoxidil lotion and topical steroid	● Hair regrowth	NR	At the end of the third session, case 1 had marked response with approximately 80% recovery of scalp hair growth ● Case 2 and case 3 demonstrated almost complete recovery of scalp hair growth after 6 and 8 sessions, respectively	No adverse effect	32 weeks
Chhabra and Verma (52)	A 11-year-old boy with AT with disease duration 1 year (scalp and eyebrow involvement)	Apremilast 10 mg twice daily for initial 10 days and the dose was increased to 30 mg morning 10 mg evening from 11th day onward with PRP injection	● Hair regrowth	NR	Hair began to regrow between 4 and 6 weeks of the therapy ● Apremilast and PRP were continued and at the end of 24 weeks robust hair growth was observed over the scalp and eyebrows except an ophiatic patch over the right temporal region which showed hair growth at 32 weeks	Mild tenderness and erythema of transient nature	32 weeks
Pototschnig and Madl (53)	A 30-year-old man with AA barbae, disease duration over 2 years	Intralesional PRP	● Hair regrowth ● Disease progression	NR	First follow up (before 2nd injection) disease was stabilized and at 1 year follow up robust regrowth was observed	Minimal discomfort within the first 36 h after injection	52 weeks
Ekelem et al. (54)	● A 60-year- old woman with patchy AA ● A 69-year-old female with AU ● A 58-year-old female with AA and FFA	Intralesional PRP	● In-line fiber-based swept-source OCT system (Thorlabs, Newton, NJ, USA) ● Hair regrowth	In-line fiber-based swept-source OCT system ● Increased in follicular unit count by week 24 in all cases	Case 1 marked clinical improvement by week 12, which was maintained or improved by week 24 ● Case 2 no clinical improvement ● Case 3 worsening at week 12 and improved by week 24	NR	24 weeks
Ederaine et al. (55)	A 31-year-old woman with AU and plaque psoriasis	Intralesional PRP with oral tofacitinib	● Hair regrowth	NR	Hair regrowth ● Significant regrowth was noted after 16 weeks of adjunctive PRP therapy	Localized pain but no major toxicities	40 weeks

AA, alopecia areata; AT, alopecia totalis; AU, alopecia universalis; FCL, fractional CO_2_ laser; FFA, frontal fibrosing alopecia; FOXP, forkhead box P3; IHC, immunohistochemistry; IL, interleukin; LLLT, low level laser therapy; NR, not reported; NSS, normal saline solution; PRP, platelet-rich plasma; SALT, severity of alopecia tool; TA, triamcinolone acetonide.

**TABLE 2:** Characteristics of primary cicatricial alopecia studies included in the systematic review.

References	Participant	Treatment	Outcome measurement	Primary outcome	Adverse effects	Duration of follow up
**Case series/case reports**						
Bolanča et al. (56)	A 25-year-old femail with LPP	Intradermal PRP injection 3 ml 4 weeks apart for 3 sessions	Not specified	● Completed regression of itching and hair shading, no perifollicular erythema and perifollicular scaling on the trichoscopy	NR	NR
Jha (57)	● Two patients with LPP (Therapeutic pearl)	Intradermal PRP injection 3 weeks apart for 4 sessions	Not specified	● Significant hair thickening	NR	NR
Özcan et al. (58)	● A 44-year-old female with FFA	Intradermal PRP injection 0.1 ml/cm2 4 weeks apart for 5 sessions in addition to TA injection, oral hydroxychloroquine, and topical minoxidil	Not specified	● After 4 weeks, perifollicular erythema, scaling, and lichenoid papules on the frontotemporal hairline were improved, and no further hair loss was noted after 20 weeks	NR	20 weeks
Jha (59)	● One patient with LPP (Therapeutic pearl)	Intradermal PRP injection 3 weeks apart for 4 sessions with topical 2% minoxidil	Not specified	● Significant hair thickening	NR	NR
Dina and Aguh (60)	● A 53-year-old woman CCCA with AGA ● A 70-year-old with LPP	Intradermal PRP injection 4–4.2 ml 4 weeks apart for 3 sessions	Follicular density at hairline	● Greater than 50% improvement in hair density along hairline ● No improvement in eyebrow lesion	NR	NR
Svigos et al. (61)	● Ten patients with FFA, FAPD, LPP 3 (30%) male, 7 (70%) female mean age 57.4 ± 15.84 years	Four PRP treatment sessions as an adjunctive	Hairline measurements from fixed points ● Trichometric measurements ● Photography	Four patients showed improvement ● Three patients showed neither improvement nor worsening ●⋅ One LPP patient showed disease progression	NR	NR
Suh et al. (62)	● A 36-year-old man with folliculitis delcavans ● A 25-year-old man with folliculitis delcavans	Intralesional PRP combined with intralesional TA at 5–6 weeks interval and oral doxycycline	Not specified	● Symptomatic improvement after 1st session, trichoscopic improvement after 3–4 sessions in both cases	NR	NR
Polster et al. (63)	● A 48-year-old patient with SLE and DLE presented with scarring alopecia	Intralesional PRP	Hair regrowth	● Significant hair regrowth was observed	NR	NR
Klein et al. (64)	● A 46-year-old woman with biopsy proven LPP resisted to conventional treatment	Intralesional PRP with oral naltrexone	Hair density ● Hair shedding	● Global improvement in hair density ●⋅ Decreased hair shedding	NR	NR

AGA, androgenic alopecia; CCCA, central centrifugal cicatricial alopecia; DLE, discoid lupus erythematous; FAPD, fibrosing alopecia in a pattern distribution; FFA, frontal fibrosing alopecia; LPP, lichen planopilaris; NR, not reported; PRP, platelet-rich plasma; SLE, systemic lupus erythematous; TA, triamcinolone acetonide.

### Platelet-rich plasma protocols

The PRP preparation protocols of included studies are demonstrated in Tables 3, 4. Regarding the centrifugation method, there were 13 studies using single spin method (33, 34, 36, 39, 43, 47, 49, 52, 53, 58, 60, 63, 64), 10 using double spin method (35, 37, 38, 40–42, 45, 46, 51, 56), and eight provided no information (44, 48, 54, 55, 57, 59, 61, 62). Several types of PRP activators were used; seven studies used calcium chloride (37, 38, 41, 45, 46, 49, 60), four used calcium gluconate (33, 34, 36, 42), one used calcium carbonate (52), two did not use any activators (48, 53), and 17 provided no information (35, 39, 40, 43, 44, 47, 51, 54–59, 61–64). The most common ratio of activator to PRP applied by the included studies was 1:9.

**TABLE 3:** Platelet-rich plasma preparation protocols of included alopecia areata studies.

References	No. of session	Interval	Max F/U	Route of application	Protocol/rpm or G—centrifuged time	Activator	Blood volume/PRP volume
**Randomized control studies**							
Trink et al. (33)	3	4 weeks	52 weeks	Intralesional injected	Single spin method ● 70 G–8 min	Calcium gluconate	36 ml/NR
El Taieb et al. (34)	3	4 weeks	12 weeks	Intralesional injection	Single spin method ● 3000 rpm–10 min	Calcium gluconate	10/4 ml
Nagaratna et al. (35)	4	3 weeks	36 weeks	Microneedling using a dermaroller then intermittent application of PRP	Double spin method ● 3000 rpm–15 min ● 2000 rpm–10 min	NR	8/5 ml
Khademi et al. (36)	4	4 weeks	16 weeks	Intralesional injection (0.1 ml/1.5–2 cm^2^)	Single spin method ● 3500 rpm–5 min	Calcium gluconate ● Calcium bicarbonate (0.1 ml per PRP 4 ml)	8/4 ml
Albalat and Ebrahim (37)	3–5	2 weeks	24 weeks	Intralesional injection (0.1 ml/cm^2^)	Double spin method ● 150 G–10 min ● 1500–2000 G–10 min	CaCl_2_ (0.1 ml of CaCl_2_ per 0.9 ml of PRP)	15/3 ml
Ragab et al. (38)	3	4 weeks	24 weeks	Group A: Intralesional PRP injection ● Group B: FCL followed by topical PRP ● Group C: microneedling by dermalroller (1.5 mm needles) followed by topical PRP	Double spin method ● 1000 rpm–15 min ● 4000 rpm–10 min	3% CaCl_2_ (0.1 ml for each 1 ml of PRP)	10 ml/NR
Kapoor et al. (39)	4	3 weeks	24 weeks	Intralesional injection (0.1 ml/cm^2^)	Single spin method/2000 rpm–3 min	NR	20 ml/NR
Hegde et al. (40)	3	4 weeks	20 weeks	Intralesional injection (0.1 ml/cm^2^)	Double spin method ● 1400 rpm–10 min ● 2800 rpm–10 min	NR	8.5 ml/NR
Balakrishnan et al. (41)	3	4 weeks	12 weeks	Intralesional injected 45° angle (0.1 ml/cm^2^)	Double spin method ● 1500 rpm–15 min ● 2500 rpm–10 min	CaCl_2_ (add CaCl_2_ 0.1 ml per 0.9 ml of PRP)	15/3 ml
Gupta et al. (42)	3	4 weeks	20 weeks	Intralesional injection (0.1 ml/cm^2^)	Manual double spin method ● 160 G–10 min ● 400 G–10 min	Calcium gluconate (1:9 ratio)	40/4–5 ml
Tawfik et al. (43)	6	1 week	18 weeks	Intralesional injection	Single spin method/3500 rpm–10 min	NR	10/5 ml
**Non-randomized studies**							
Singh (44)	6	4 weeks	52 weeks	Intralesional injection	NR	NR	25 ml/NR
Khan et al. (45)	3	4 weeks	36 weeks	Intralesional injection (0.1 ml/cm^2^ at a site 1 cm apart)	Takikawa’s manual double spin method with slight modification ● 2000 rpm–10 min ● 4000 rpm–10 min	10% CaCl_2_ (0.3 ml for 1 ml of PRP)	15 ml/NR
Fayed et al. (46)	Ten or shorter if hair regrowth occurred	2 weeks	20 weeks	Intralesional injection (20 mm apart)	Double spin method ● 3000 rpm–7 min ● 4000 rpm–5 min	CaCl_2_	10–30 ml/NR
Fawzy et al. (47)	3	4 weeks	12 weeks	Intralesional injection (0.1 ml/cm^2^)	Single spin method ● 3000 rpm–10 min	NR	10/2–3 ml
**Case series/Case reports**							
Donovan (48)	1	–	12 weeks	Intralesional injection	NR	Not used	120/9 ml
Mubki (49)	4	2 weeks	18 weeks	Intralesional injection (0.1 ml/cm^2^)	Single spin method ● 1500 G–4 min	CaCl_2_	18 ml/NR
De Vasconcelos et al. (50)	3	3 weeks	9 weeks	Intralesional injection (2 cm apart)	NR	NR	NR
Fonseka et al. (51)	Until a satisfactory response was obtained	4 weeks	32 weeks	Intralesional injection (1 cm apart)	Double spin method ● 3000 rpm–10 min ● 2000 rpm–5 min	NR	40/10 ml
Chhabra and Verma (52)	NR	2 weeks	32 weeks	Intralesional injection (0.1 ml/cm^2^)	Single spin method ● 3000 rpm–10 min	Calcium carbonate 0.1 ml	20 ml/NR
Pototschnig and Madl (53)	3	6 weeks	52 weeks	Intralesional injection (1 cm apart at a depth of 2–3 mm)	Single spin method ● 350 G–5 min	Not used	30 ml/NR
Ekelem et al. (54)	3	6 weeks	24 weeks	Intralesional injection	NR	NR	NR/NR
Ederaine et al. (55)	NR	4 weeks	40 weeks	Intralesional injection	NR	NR	NR/5 ml

G, gravitational force; NR, not reported; PRP, platelet rich plasma; rpm, revolutions per minute.

**TABLE 4:** Platelet-rich plasma preparation protocols of included primary cicatricial alopecia studies.

References	No. of session	Interval	Max F/U	Route of application	Protocol/rpm or G	Activator	Blood volume/PRP volume
**Case series/case reports**							
Bolanča et al. (56)	3	4 weeks	12 weeks	Intralesional injection	Double spin method ● 500 G–10 min ● 1520 G–10 min	NR	15/3 ml
Jha (57)	4	3 weeks	12 weeks	Intralesional injection	NR	NR	NR/NR
Özcan et al. (58)	5	4 weeks	20 weeks	Intralesional injection (0.1 ml/cm^2^)	Single spin method ● 4000 rpm–10 min	NR	14 ml/NR
Jha (59)	4	3 weeks	12 weeks	Intralesional injection	NR	NR	NR/NR
Dina and Aguh (60)	3	4 weeks	12 weeks	NR	Single spin method ● 1100 G–6 min	0.5-M CaCl_2_	9/4–4.2 ml
	3	4 weeks	12 weeks	NR	Single spin method ● 1100 G–6 min	0.5-M CaCl_2_	9/4–5 ml
Svigos et al. (61)	Until disease stable	NR	NR	Intralesional injection	NR	NR	NR/NR
Suh et al. (62)	3	4 weeks	12 weeks	NR	Single spin method ● 1100 G–6 min	0.5-M CaCl_2_	9/4–5 ml
	3	6–9 weeks	20 weeks	NR	NR	NR	NR/NR
Polster et al. (63)	3	12 weeks	36 weeks	Intralesional injection	Single spin method ● 3500 rpm–10 min	NR	22/7 ml
Klein et al. (64)	3	4 weeks	12 weeks	Intralesional injection (0.1 ml/cm^2^)	Single spin method	NR	NR/5 ml

CaCl_2_, calcium chloride; FCL, fractional CO_2_ laser; G, gravitational force; NR, not reported; PRP, platelet rich plasma; rpm, revolutions per minute.

Regarding treatment protocol, most studies used three or four treatment sessions, with 15 using three sessions (33, 34, 38, 40–42, 45, 47, 50, 53, 54, 56, 60, 63, 64), six using four sessions (35, 36, 39, 49, 57, 59), two treated until a satisfactory response was obtained (51, 61), and the remaining studies used different number of sessions. Our included studies selected different treatment intervals, with the most common interval being 4 weeks, selected by 17 studies (33, 34, 36, 38, 40–42, 44, 45, 47, 51, 55, 56, 58, 60, 62, 64); five studies selected 3 weeks (35, 39, 50, 57, 59), four selected 2 weeks (37, 46, 49, 52), one selected 1 week (43), and four selected interval of ≥ 6 weeks (53, 54, 62, 63).

### Efficacy of platelet-rich plasma for alopecia areata

#### Platelet-rich plasma monotherapy for alopecia areata

Several studies have demonstrated hair regrowth in AA lesions after PRP monotherapy. Among studies included, PRP showed superior efficacy compared to placebo in Severity of Alopecia Tool (SALT) score reduction (35, 42, 46), hair regrowth (35, 42, 45), and decrease in dystrophic hairs (42, 54), in mild cases of AA regardless of disease duration. For more severe AA cases, Khademi et al. found that PRP as monotherapy was relatively ineffective in alopecia totalis (AT) (36). Regarding different delivery methods of PRP, Ragab et al. reported that the efficacy of PRP in SALT score reduction was comparable among intradermal injection, fractional CO2 laser, and microneedling. At the end of the study, no significant difference between the groups was observed in physician clinical assessment and patient satisfaction (38).

#### Platelet-rich plasma compared with other treatments for alopecia areata

Clinical trials were conducted to compare PRP with intralesional triamcinolone acetonide (TA). PRP was found to be non-inferior to intralesional TA, a standard treatment for patchy AA. Trink et al. compared PRP to 2.5 mg/ml intralesional TA and found that PRP therapy resulted in a greater reduction in SALT score and improved dermoscopic features and relapse rates (33). According to studies comparing higher TA concentrations (10 mg/ml) with PRP in which most patients had patchy AA with < 25% scalp involvement or < 6 months of disease duration, each treatment had comparable efficacy (37, 39–41, 47). However, according to a few studies, TA-treated groups demonstrated a greater reduction in SALT score and greater hair regrowth (39, 40). Two studies found that the PRP group had a lower relapse rate than the corticosteroid group (33, 37). Efficacy of PRP in AA has also been compared to topical minoxidil and low-level laser therapy (LLLT). PRP showed superior to 5% topical minoxidil in improving dystrophic hair and had a greater effect on improving hair diameter compared to LLLT (34, 43).

#### Platelet-rich plasma as an adjunctive treatment for alopecia areata

Studies investigating PRP as a co-intervention for AA are limited. Mubki reported an increased hair diameter in combined PRP and TA injected scalp side compared to a decline in the contralateral side in a 22-year-old female with chronic diffuse AA for 5 years (49). Two case reports published in 2019 reported some efficacy of PRP as adjuvant therapy on hair regrowth in AT patients (51, 52). Of the two studies, one study initiated PRP as adjuvant therapy after a 7-month course of Janus kinase inhibitor (JAKi) treatment in an 11-year-old patient with AT (52), and another study added PRP as an adjuvant to topical corticosteroids and minoxidil (51). In addition, Ederaine et al. reported an adjuvant effect of PRP with JAKi, showing significant hair regrowth after 4 months of combined treatment in a 31-year-old woman with plaque psoriasis who presented with patchy AA progressed to alopecia universalis (AU) (55).

### Efficacy of platelet-rich plasma for primary cicatricial alopecias

There are only a few studies that documented the efficacy of PRP in PCAs to date. In our review, six case reports and three case series addressed the efficacy of PRP in hair regrowth, reduction of clinical itching and scaling, and improvement of dermoscopic features (perifollicular erythema and scaling) after an average of three PRP sessions (56–64). Among them, two case series demonstrated a more reliable perspective of PRP efficacy. One case series comprising 10 patients showed variable treatment responses depending on patients’ characteristics (61), and another, comprising two patients, indicated decreasing efficacy of PRP treatment over time (60). Patients in included studies had lymphocytic (i.e., lichen planopilaris, frontal fibrosing alopecia, fibrosing alopecia in a patern distribution, discoid lupus erythematosus, and central centrifugal cicatricial alopecia) and neutrophilic PCAs (i.e., folliculitis decalvans). Four of the reported efficacious studies used intradermal PRP injection as monotherapy, and patients in two of four studies had concomitant androgenetic alopecia (56, 57, 60, 63).

### Quality assessment

**FIGURE 2:**
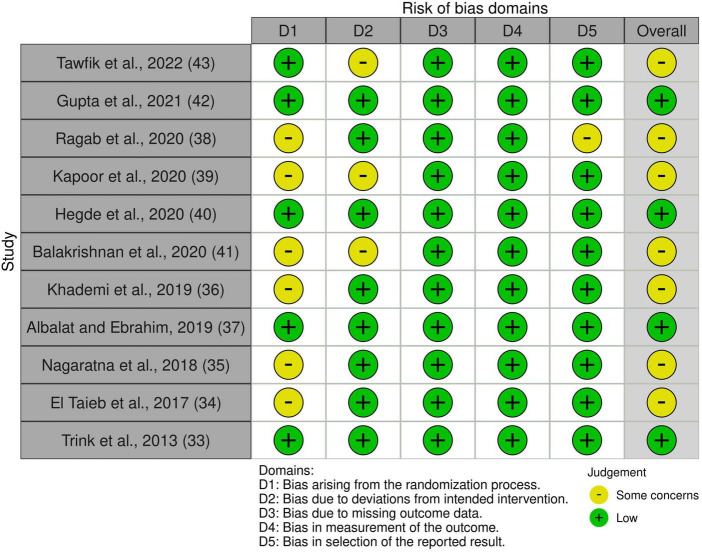
Risk of bias of included non-randomized studies.

**FIGURE 3:**
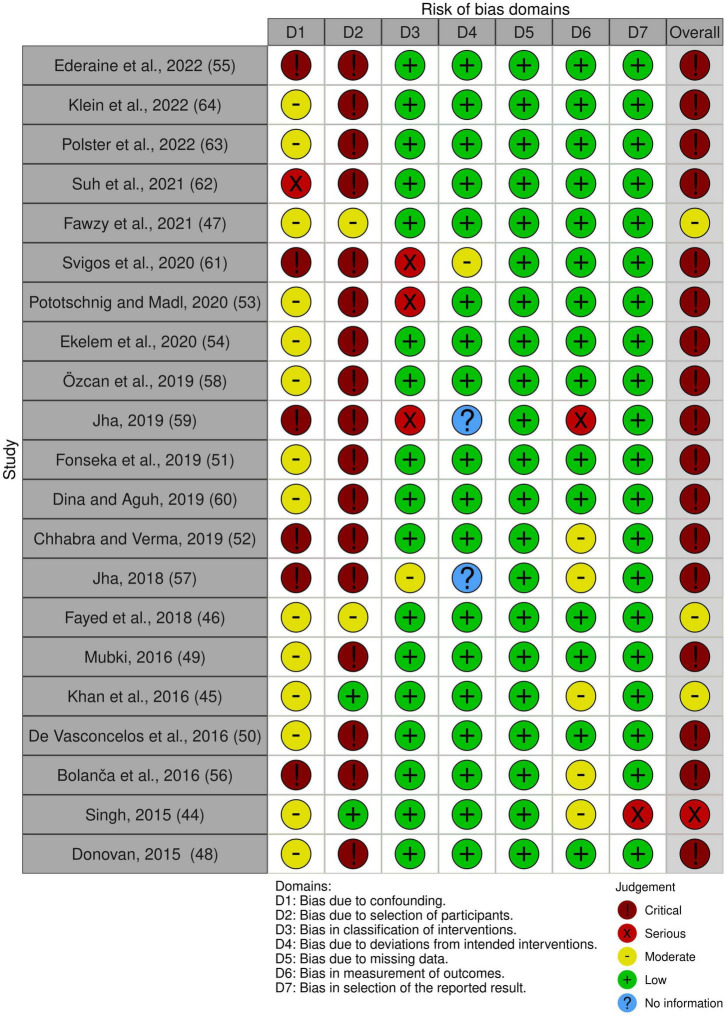
Risk of bias of included randomized studies.

Non-randomized studies, particularly case reports and case series, were rated as having a serious or critical risk of bias, mainly due to their inherent potential for confounding and selection bias. All RCTs included were rated as having either low risk or some concerns for overall bias. Risk-of-bias plots are shown in Figures 2, 3.

## Discussion

Increasing evidence emphasizes the efficacy of PRP in treating IMAs. The present systematic review has retrieved a sufficient number of available clinical trials regarding PRP treatment for AA and PCAs to perform a pertinent systematic analysis of results. Our study demonstrates promising results for PRP treatment of patch-type AA either as monotherapy or when compared to intralesional TA, topical minoxidil, and LLLT. Moreover, our analysis reveals the efficacy of PRP treatment for PCAs in case reports and small case series. Nevertheless, cumulative evidence is not as convincing for PRP use as standard treatment for AA and PCAs.

PRP therapy is a novel technique comprising autologous plasma preparations with concentrated platelets. Its regenerative effects are gaining momentum in hair loss treatment. PRP is a promising treatment for IMAs because it uses the patient’s healing mechanism, acting on multiple biological targets with minimal immune reaction concerns (27, 29). Nevertheless, how PRP elicits therapeutic effects in IMAs remains unclear. Based on current evidence, PRP helps regenerate and repair HFs by releasing several key growth factors and cytokines (e.g., PDGF, FGF, EGF, and IGF) that play critical roles in HF stem cell differentiation and proliferation (26, 27). Additionally, PRP impacts the anti-inflammatory effect by downregulating monocyte chemoattractant protein-1, matrix metalloproteinase (MMP)-3, MMP-13, and a disintegrin and metalloproteinase with thrombospondin motifs-5, and immunomodulatory properties by upregulating IL (interleukin)-4, IL-10, IL-13, and TGF-β (65–68). Furthermore, PRP may restore normal skin, prevent fibrosis, and remodel scar tissue (69–72).

Our analysis reveals that PRP has demonstrated favorable results in treating IMAs. The data support the use of PRP as a promising, safe, office-based therapy for hair regrowth in patients with patchy AA; however, variable responses were reported in severe AA types, including AT and AU. Most RCTs demonstrate comparable PRP efficacy to intralesional TA with earlier and more persistent responses (37, 39–41, 47). PRP also showed superior efficacy compared to 5% topical minoxidil and LLLT (34, 43). In contrast to AA, PCAs have fewer studies evaluating the efficacy of PRP, and their treatment endpoint is disease stabilization. Patients in included studies had lymphocytic and neutrophilic scarring alopecias. Case reports and small case series have shown positive clinical outcomes (56–60, 62–64), whereas one case series revealed variable efficacy of PRP treatment (61). However, the use of PRP to treat IMAs is at the initial stage, and several issues remain to be addressed, including efficacy in more severe forms of AA and other subtypes of PCAs, PRP safety, and standard protocols.

PRP is a relatively safe procedure with mild adverse effects, such as tolerable pain, scalp discomfort, burning sensation, and transient erythema. To date, there have been no reports of serious adverse events, such as bleeding and infection. Nevertheless, all included studies highlighted the safety of PRP for IMAs only in short follow-up duration, which could not support its safety appropriately. Notably, contraindications for PRP treatment include hemodynamic instability, coagulation disorders, and infection at the treated site (73).

Although PRP is effective in many studies, its clinical application is complicated by the lack of consensus regarding its preparation and treatment protocol given the number of variables, including equipment, centrifugation forces, number and length of centrifugation, number and interval of treatment sessions, and dosage. Furthermore, evidence supporting long-term maintenance and criteria for treatment candidates is still lacking. The heterogeneity in PRP therapy requires further well-designed studies to overcome these surrounding controversies.

Moreover, it is difficult to determine whether the efficacy of PRP is due to the growth factors and cytokines within the PRP or their production as a result of needle injection-induced trauma since there is currently no solid evidence to support the mechanism of PRP for treating hair disorders speculated by previous studies. There are conflicting results in included studies with a split-scalp design comparing PRP with normal saline solution (36, 42) or TA (33, 40) injections. Previous RCT comparing the efficacy of PRP vs. saline in 26 patients with androgenic alopecia found it an effective treatment; however, the growth factor levels (i.e., PDGF, EGF, and VEGF) did not correlate with clinical improvement (74). The mechanism responsible for improvement following PRP injection remains to be investigated.

This systematic review has some limitations. We included all types of study designs, which contained bias-prone case series and case reports in our analysis. As a result, many of the included studies are of poor quality, particularly those on PCAs. Moreover, many studies have small sample sizes. Lastly, the high heterogeneity between studies, such as diverse PRP preparation, outcome evaluation methods, and disease severity of study populations, prohibits quantitative analysis.

## Conclusion

This systematic review reports preliminary evidence that PRP is a promising treatment option for IMAs, particularly in individuals who fail conventional therapies, experience adverse effects, or are contraindicated for other modalities. PRP is a relatively effective treatment for regrowing hairs in AA and alleviating disease progression in PCAs with minimal adverse effects. However, this conclusion is mostly based on limited evidence, including case reports and series and studies with small sample sizes without a proper control group. Moreover, standardized protocols for PRP preparation and treatment remain controversial. Further large-scale, high-quality RCTs with a longer duration of follow-up are crucial to confirm the efficacy and safety of PRP in IMAs. Currently, there is insufficient evidence to support using PRP as standard treatment.

## Data availability statement

The original contributions presented in this study are included in the article/Supplementary material, further inquiries can be directed to the corresponding author.

## Author contributions

PS: conceptualization and writing—review and editing. PS, KT, and TY: methodology. PS and NS: validation. KT and TY: formal analysis. KT, TY, and NS: investigation and writing—original draft preparation. NS: data curation. All authors have read and agreed to the published version of the manuscript.
