# Supplementary material for: Platelet-rich plasma in alopecia areata and primary cicatricial alopecias: A systematic review
Source: Front Med (Lausanne). 2022 Nov 24;9:1058431. doi: 10.3389/fmed.2022.1058431 (PMC9731377; doi:10.3389/fmed.2022.1058431)
Supplement: Supplementary file 1 [file Table_1.DOCX]

**Supplemental Table 1.** Details of the search strategy and search terms**.**

| **PubMed** | ("platelet-rich plasma"[MeSH Terms] OR “platelet-rich plasma” OR “platelet rich plasma” OR “PRP”) AND (“alopecia areata” [MeSH Terms] OR “alopecia areata” OR “lichen planopilaris” OR “frontal fibrosing alopecia” OR “scarring alopecia” OR “cicatricial alopecia”) |
| --- | --- |
| **Embase** | 1. 'platelet rich plasma'/exp OR 'platelet rich plasma' OR 'prp' 2. 'alopecia areata'/exp OR 'alopecia areata' 3. 'lichen planopilaris'/exp OR 'lichen planopilaris' 4. 'frontal fibrosing alopecia'/exp OR 'frontal fibrosing alopecia' 5. 'cicatricial alopecia'/exp OR 'cicatricial alopecia' OR 'scarring alopecia'/exp OR 'scarring alopecia') 6. 2 OR 3 OR 4 OR 5 7. 1 AND 6 |
| **Scopus** | 1. TITLE-ABS-KEY ( "platelet-rich plasma" OR "platelet rich plasma" OR "prp" ) 2. TITLE-ABS-KEY ( "alopecia areata" OR "lichen planopilaris" OR "frontal fibrosing alopecia" OR "scarring alopecia" OR "cicatricial alopecia" ) 3. 1 AND 3 |
| **Cochrane Library** | 1. MeSH descriptor: [Platelet-Rich Plasma] explode all trees 2. MeSH descriptor: [Alopecia Areata] explode all trees 3. "Lichen planopilaris" 4. "Frontal fibrosing alopecia" 5. "Scarring alopecia" 6. "Cicatricial alopecia" 7. #2 OR #3 OR #4 OR #5 OR #6 8. #1 AND #7 |
